# 儿童造血干细胞移植后水痘-带状疱疹病毒感染临床分析

**DOI:** 10.3760/cma.j.issn.0253-2727.2022.05.014

**Published:** 2022-05

**Authors:** 琳 童, 璐颖 张, 岩 孟, 贤敏 管, 洁 于, 颖 窦

**Affiliations:** 重庆医科大学附属儿童医院血液肿瘤科，国家儿童健康与疾病临床医学研究中心，儿童发育疾病研究教育部重点实验室，儿童感染免疫重庆市重点实验室，重庆 400014 Department of Hematology Oncology Children's Hospital of Chongqing Medical University, National Clinical Research Center for Child Health and Disorders, Ministry of Education Key Laboratory of Child Development and Disorders, Chongqing Key Laboratory of Child Infection and Immunity, Chongqing 400014, China

造血干细胞移植（HSCT）是治疗血液系统恶性疾病、大部分原发性免疫缺陷病以及部分遗传代谢性疾病的有效措施[1]。但患者移植后面临着潜伏的既往感染的病毒再激活或新发感染的风险。水痘-带状疱疹病毒（VZV）属ɑ-疱疹病毒，是人类特有的嗜神经病毒，原发感染后可长期潜伏于神经节细胞中，免疫功能下降、疾病等因素可再激活VZV，引起带状疱疹后遗神经痛、眼部病变、脊髓炎、脑膜炎、脑炎、血管病变等并发症[2]。为了解儿童HSCT后VZV感染的发病情况，我们回顾性分析18例HSCT后发生VZV感染患儿的临床特征、相关危险因素及诊疗预后情况。

## 病例与方法

1. 病例资料：回顾性分析2007年1月至2020年10月于重庆医科大学附属儿童医院血液肿瘤科行HSCT的360例患儿，所有病例均为首次移植。其中男265例（73.6％），女95例（26.4％），中位年龄3（0.3～17）岁。白血病、再生障碍性贫血、地中海贫血、骨髓增生异常综合征等造血系统疾病177例（49.2％），原发性免疫缺陷病172例（47.8％），淋巴系统疾病（包括各种淋巴瘤、噬血细胞综合征等）9例（2.5％），神经母细胞瘤2例（0.6％）。移植情况：allo-HSCT 351例（97.5％），其中183例为HLA全相合移植，168例为HLA不全相合移植；auto-HSCT 9例（2.5％）。移植物类型：外周血311例（86.4％），骨髓8例（2.2％），脐血36例（10.0％），外周血+骨髓5例（1.4％）。急性移植物抗宿主病（aGVHD）的诊断符合改良Glucksberg分级分度标准，慢性移植物抗宿主病（cGVHD）的诊断符合NIH分级标准。

2. 移植前预防性抗病毒治疗情况：360例患儿中，9例（2.5％）移植前1个月内未使用抗病毒药物（主要分布于2007-2013年），其余351例（97.5％）均在预处理前1周常规预防性使用抗病毒药物。320例（88.9％）接受阿昔洛韦治疗，其中46例20～40 mg·kg^−1^·d^−1^口服给药，274例10 mg/kg每8 h 1次静脉给药。29例（8.1％）因合并巨细胞病毒感染使用更昔洛韦5 mg/kg每12 h 1次静脉滴注。1例（0.3％）利巴韦林15 mg·kg^−1^·d^−1^静脉给药，1例（0.3％）因合并乙肝病毒感染使用恩替卡韦0.25 mg/d口服给药。

3. VZV感染的定义与诊断：VZV感染主要为临床诊断，定义为出现特征性水疱疹和（或）以下实验室检测存在阳性发现者：①疱疹液VZV-PCR阳性；②血液VZV-IgM抗体阳性；③血液VZV病毒载量高于正常上限（正常参考值<400 拷贝数/ml）。所有感染VZV的患儿均根据病史、症状体征、辅助检查结果及经过我院皮肤科与感染科会诊后确诊。重症病例定义为出血性水痘和（或）存在VZV感染并发症。

4. VZV感染并发症的诊断与鉴别：VZV感染可合并血小板减少、紫癜、血尿、胃肠出血、肺炎、肾炎、心肌炎、脑炎、继发细菌感染等多器官系统并发症，并发症的诊断除出现相应症状体征外，还需与VZV皮疹出现存在时间关联，且予以常规抗GVHD治疗无效而抗病毒治疗有效。此外，脑炎的诊断还需满足脑脊液VZV-DNA检测阳性，VZV导致的肺炎其影像学呈典型的间质性肺炎并双侧弥漫性结节致密影或网织状阴影，胃肠道并发症需首先除外肠道细菌感染，加之一线及二线抗GVHD无效方可考虑，必要时行胃镜和肠镜下黏膜活检以鉴别肠道aGVHD。

5. 统计学处理：数据统计分析采用SPSS 26.0统计软件包进行处理，单因素分析采用*χ*^2^检验或Fisher精确概率法，根据单因素分析结果用二分类Logistic回归进行多因素分析。*P*<0.05为差异有统计学意义。

## 结果

1. HSCT术后感染VZV患儿临床特征及感染治疗情况：360例患儿中18例HSCT后发生VZV感染，发生率为5.0％，男女比为2∶1，中位年龄9（2～15）岁，其中水痘4例（22.2％）、带状疱疹14例（77.8％），中位发生时间为移植后98（22～474）d，移植后100 d内发病9例（50.0％），移植后1年内发病16例（88.9％）（表1）。18例患儿中7例在移植前接种过水痘疫苗（38.9％），均在服用免疫抑制剂时发病，主要表现为皮肤散在分布的粟粒样红色丘疱疹，部分融合成片，或簇集分布的针尖样细小水疱，部分伴瘙痒及疼痛，其中3例为出血性水痘（16.7％），5例发生并发症（27.8％），包括病毒性脑炎（例3）、消化道出血（例9）、肺出血（例11）、Ramsay-Hunt综合征（例15）及继发皮肤细菌感染（例11、例17），因此共计7例为重症VZV感染（38.9％）。此外，这些患儿中仅1例（5.6％）在移植后有新近水痘接触史（例5），共7例（38.9％）存在移植前明确水痘感染或接触史，其中1例来源于供者（例12），另有1例（5.6％）为既往可疑带状疱疹病史（例3），而此8例患儿移植后再激活时均表现为带状疱疹。治疗上，所有患儿均采用丙种球蛋白输注、阿昔洛韦静脉滴注、调整免疫抑制剂用量及辅以外用药物的方案，继发皮肤细菌感染的加用阿米卡星外用，中位治疗时间12（7～120）d，出院时皮疹均消退且达到临床痊愈标准，无1例因感染VZV而发生死亡。例15第1次发病为术后2个月（带状疱疹并发Ramsay-Hunt综合征），治疗15 d后好转，出院约4个月后再次出现皮肤散在疱疹，无并发症，予阿昔洛韦抗感染治疗7 d后皮疹明显消退好转。此外，有1例患儿（例11）治疗时间远超其他病例（120 d），该患儿出院1周后腹壁出现新发丘疹继发细菌感染，治疗3个月后皮疹消退并结痂。

**表1:** 18例造血干细胞移植后水痘-带状疱疹病毒感染患儿的一般资料

例号	性别	移植年龄	原发病	移植类型	供者来源	HLA相合度	预处理方案	抗感染方案	抗GVHD方案	GVHD
1	男	2岁7月	WAS	UCBT	无关供者	6/6全相合	BU+CY	IPM+KETOC+MCR+ACV	CsA+MPED	Ⅰ度aGVHD（已控制）
2	男	12岁10月	Common B-ALL	PBSCT	同胞弟弟	10/10全相合	BU+CY	IPM+NOR+ACV	CsA+MTX	无
3	男	4岁6月	WAS	PBSCT	无关供者	10/10全相合	BU+CY	IPM+NOR+AZM+ACV	CsA+MTX+PDN	Ⅰ~Ⅱ度aGVHD（已控制）
4	男	3岁7月	WAS	UCBT	无关供者	9/10相合	BU+CY+VP16	IPM+MCM+ACV	CsA+MTX	Ⅰ度aGVHD（已控制）
5	男	3岁10月	WAS	UCBT	无关供者	8/10相合	Flu+BU+ATG	IPM+MCM+GCV	CsA+MMF	Ⅱ度aGVHD（已控制）
6	男	10岁4月	AWLL	PBSCT	同胞弟弟	10/10全相合	Flu+BU+CY	IPM+MCM+ACV	CsA	无
7	女	8岁8月	AA	PBSCT	父女	8/10相合	BU+CY+Ara-C	IPM+KETOC+ACV	CsA+MMF	Ⅰ度aGVHD（已控制）
8	女	13岁6月	Common B-ALL	PBSCT	同胞哥哥	10/10全相合	Flu+BU+CY+ATG	IPM+MCM+ACV	CsA+MTX	cGVHD（部分控制）
9	男	9岁10月	T-ALL	PBSCT	无关供者	8/10相合	BU+CY+VP16	IPM+MCM+ACV	CsA+MTX+FK506	Ⅱ度aGVHD（基本控制）
10	男	3岁7月	MDS	PBSCT	无关供者	9/10相合	BU+CY+Ara-C+ATG	IPM+MCM+ACV	CsA+MTX	Ⅰ度aGVHD（已控制）
11	男	14岁7月	T-ALL	PBSCT	无关供者	10/10全相合	BU+CY+VP16+ATG	IPM+MCM+ACV	CsA+MTX	Ⅱ度aGVHD（已控制）
12	男	8岁8月	AA	PBSCT	同胞姐姐	10/10全相合	Flu+CY+ATG	IPM+MCM/CAS+ACV	CsA+MTX	Ⅰ度aGVHD（已控制）
13	女	1岁10月	β-地中海贫血	PBSCT	无关供者	9/10相合	Flu+BU+CY+ATG	IPM+CAS+ACV	MMF+MTX	Ⅰ度aGVHD（已控制）
14	女	14岁3月	Common B-ALL	PBSCT	无关供者	9/10相合	BU+CY+VP16+ATG	IPM+KETOC+ACV	CsA+MTX	Ⅱ度aGVHD（部分控制）
15	男	9岁3月	AA	PBSCT	无关供者	9/10相合	Flu+BU+CY+ATG	IPM+KETOC+ACV	CsA+MMF	Ⅱ度aGVHD（部分控制）
16	男	12岁10月	APDS	PBSCT	同胞姐姐	10/10全相合	低强度Flu+BU	IPM+SCF+KETOC+ACV	CsA+MTX	Ⅰ度aGVHD（尚未控制）
17	女	3岁2月	Pre B-ALL	PBSCT	父女	7/10相合	BU+CY+VP16+ATG	IPM+LEV+KETOC+ACV	CsA+MMF+MTX	Ⅰ度aGVHD（已控制）
18	女	11岁6月	MDS	PBSCT	同胞哥哥	10/10全相合	BU+CY+Ara-C	IPM+LEV+KETOC+ACV	CsA+MTX	无

注：WAS：Wiskott-Aldrich综合征；common B-ALL：急性普通型B淋巴细胞白血病；AWLL：急性混合细胞性白血病；AA：再生障碍性贫血；T-ALL：急性T淋巴细胞白血病；MDS：骨髓异常增生综合征；APDS：PI3Kδ过度活化综合征；Pre B-ALL：急性前B淋巴细胞白血病；UCBT：脐血移植；PBSCT：外周血干细胞移植；BU：白消安；CY：环磷酰胺；VP16：依托泊苷；Flu：氟达拉滨；ATG：抗人胸腺细胞免疫球蛋白；Ara-C：阿糖胞苷；IPM：亚胺培南；KETOC：伏立康唑；MCR：小诺霉素；ACV：阿昔洛韦；GCV：更昔洛韦；NOR：诺氟沙星；AZM：阿奇霉素；MCM：米卡芬净；CAS：卡泊芬净；SCF：头孢哌酮/舒巴坦；LEV：左氧氟沙星；CsA：环孢素A；MPED：甲泼尼龙；MTX：甲氨蝶呤；PDN：泼尼松；MMF：霉酚酸酯；FK506：他克莫司；aGVHD：急性移植物抗宿主病；cGVHD：慢性移植物抗宿主病

**表2:** 18例造血干细胞移植后水痘-带状疱疹病毒感染患儿的感染及治疗情况

例号	发病时间（d）	相关既往史	水痘疫苗接种史	发病类型	受累部位	并发症	合并病毒感染	治疗时间（d）	转归	复发情况
1	81	无	未接种	水痘	皮肤	无	无	12	好转	无
2	22	既往水痘病史	未接种	带状疱疹	皮肤^a^	无	无	18	好转	无
3	136	既往可疑带状疱疹病史	未接种	带状疱疹	皮肤、中枢神经系统	病毒性脑炎	无	14	好转	无
4	211	既往水痘病史	未接种	带状疱疹	皮肤	无	CMV、EBV	14	好转	无
5	34	新近水痘接触史	移植前接种	水痘	皮肤	无	CMV、EBV、RSV	12	好转	无
6	23	无	未接种	带状疱疹	皮肤	无	无	12	好转	无
7	356	既往水痘接触史	移植前接种	带状疱疹	皮肤	无	无	8	好转	无
8	442	无	移植前接种	带状疱疹	皮肤	无	CMV	13	好转	无
9	89	无	未接种	水痘	皮肤^a^、消化道	消化道出血	CMV、BKV	7	好转	无
10	474	既往水痘病史	移植前接种	带状疱疹	皮肤	无	无	9	好转	无
11	67	既往水痘病史	未接种	带状疱疹	皮肤、肺部	肺出血、继发皮肤感染	CMV、EBV、BKV	120	好转	无
12	45	供者既往水痘病史	未接种	带状疱疹	皮肤	无	无	11	好转	无
13	195	无	未接种	带状疱疹	皮肤	无	EBV、CMV	7	好转	无
14	110	无	移植前接种	水痘	皮肤^a^	无	EBV、CMV	9	好转	无
15	62	无	未接种	带状疱疹	皮肤、面神经	Ramsay-Hunt综合征	无	15	好转	复发1次
16	107	既往水痘病史	移植前接种	带状疱疹	皮肤	无	无	8	好转	无
17	116	无	未接种	带状疱疹	皮肤	继发皮肤感染	无	10	好转	无
18	76	无	移植前接种	带状疱疹	皮肤	无	无	15	好转	无

注：CMV：巨细胞病毒；EBV：EB病毒；RSV：呼吸道合胞病毒；BKV：BK病毒；a：出血性水疱疹

2. HSCT术后患儿VZV感染的危险因素分析：单因素分析结果显示HSCT术后是否发生VZV感染与移植受者性别、原发疾病类别、HLA相合、供受者关系、干细胞来源均无显著相关性（*P*>0.05），与患儿年龄有关（*P*＝0.001）（表3）。

**表3:** 影响儿童造血干细胞移植后水痘-带状疱疹病毒（VZV）感染的单因素分析［例（％）］

因素	感染VZV（18例）	未感染VZV（342例）	*χ^2^*值	*P*值
患儿性别			0.169	0.681
男	12（4.5）	253（95.5）		
女	6（6.3）	89（93.7）		
患儿年龄			10.700	0.001
<6岁	7（2.6）	261（97.4）		
≥6岁	11（12.0）	81（88.0）		
原发病类型				0.247
原发性免疫缺陷病	5（2.9）	167（97.1）		
造血系统疾病	13（7.3）	164（92.7）		
淋巴组织疾病	0（0）	9（100.0）		
恶性实体瘤	0（0）	2（100.0）		
HLA相合度			0.085	0.771
全相合	9（4.7）	183（95.3）		
不全相合	9（5.4）	159（94.6）		
移植类型				1.000
allo-HSCT	18（5.1）	333（94.9）		
auto-HSCT	0（0）	9（100.0）		
干细胞来源				0.703
外周血	15（4.8）	296（95.2）		
骨髓	0（0）	8（100.0）		
脐血	3（8.3）	33（91.7）		
外周血+骨髓	0（0）	5（100.0）		

3. 水痘疫苗接种情况与移植后VZV感染严重程度的相关性分析：移植前水痘疫苗接种与否与移植后VZV感染的严重程度（*P*＝0.151）、是否发生并发症（*P*＝0.101）、是否复发（*P*＝1.000）均无显著相关性（表4）。

**表4:** 水痘疫苗接种与否与移植后水痘-带状疱疹病毒（VZV）感染情况相关性分析［例（％）］

水痘疫苗接种情况	例数	VZV感染严重程度		发生并发症		复发	
		轻症	重症	是	否	是	否
接种	7	6（85.7）	1（14.3）	0（0.0）	7（100.0）	0（0.0）	7（100.0）
未接种	11	5（45.5）	6（54.5）	5（45.5）	6（54.5）	1（9.1）	10（90.1）

*P*值		0.151		0.101		1.000

## 讨论

儿童HSCT后VZV感染发生率为7.3％～15.2％[3]–[6]，而本研究结果显示为5.0％，其差异可能来源于三个方面：一是各研究病例来源、组成不一，患儿原发疾病与术前免疫水平可能对术后病毒的再激活产生影响；二是潜在的失访，如患儿感染后未至我院就诊、早期病例资料缺失等；三是本研究97.5％的患儿移植前常规使用阿昔洛韦或更昔洛韦预防病毒感染，可能降低了VZV再激活率。既往多项研究[7]–[9]显病毒感染，可能降低了VZV再激活率。既往多项研究[7]–[9]显示，未预防性使用抗病毒药物的情况下，25％～32％的患儿在移植后发生VZV感染。近年来随着病毒预防方案的广泛应用，VZV感染发生率已大幅降低。本组病例VZV感染中位发生时间为移植后98（22～474）d，大部分（77.8％）患儿于移植后30 d～1年内发病，发病时间相对较晚，与文献[6]报道相符，反映了T淋巴细胞的功能尚不成熟。

本研究通过危险因素分析发现HSCT后VZV感染发病与年龄有关，≥6岁患儿术后VZV感染发生率显著高于<6岁患儿。国内外多项文献均发现HSCT后VZV感染发病具有年龄依赖性，发病率随年龄增长而升高[5]–[6]，这提示我们对于移植后的年长儿需格外关注既往有无水痘病史，以及近期有无水痘或带状疱疹患者接触史，必要时或可预防性使用阿昔洛韦以降低感染风险。本研究数据显示移植前患儿接种水痘疫苗与否与术后VZV感染的严重程度、是否发生并发症、是否复发均无显著相关，但由于本研究移植后发生VZV感染的病例数较少，此结论还需扩大样本量进一步验证。

为预防VZV感染，有研究人员建议VZV血清学阴性的患者在HSCT前至少4周接受VZV疫苗接种，但不应早于完成化疗疗程后3个月或至少在抗B细胞抗体治疗后12个月，也有观点认为移植患者在移植后接种VZV疫苗是最明智的，但其有效性、术后接种策略及对病毒再激活的影响仍需进一步研究[10]–[11]。美国感染性疾病协会（IDSA）2013年推荐[12]满足以下条件的HSCT患者可接受2剂水痘疫苗接种：①年龄≥12月；②VZV血清学阴性；③移植预处理前至少4周或移植后至少24个月且未发生GVHD；④未处于免疫抑制状态；12岁以下患儿接种第2剂与第1剂至少间隔3个月，13岁以上至少间隔4周。

综上所述，本研究发现VZV感染及再激活是儿童HSCT术后常见的并发症之一，多在移植后中晚期出现，且具有年龄依赖性，因此年长患儿需格外关注VZV感染相关病史。本研究不足之处在于，一方面纳入的阳性病例数较少，具有样本量的局限性；另一方面本研究为回顾性研究，资料较为有限，部分久远病例随访困难，影响因素混杂且不可控，因此可设计前瞻性研究探索水痘疫苗接种对移植后发生VZV感染的预防及保护作用，并探索最佳接种时机，如何制定最佳的预防性抗病毒方案也亟需深入研究，从而在多个环节预防VZV感染，提高患儿移植后生存质量及临床预后。
